# Retraction: ABO-incompatible liver transplantation for severe hepatitis B patients

**DOI:** 10.3389/ti.2026.17398

**Published:** 2026-07-27

**Authors:** Transplant International Editorial Office

Transplant International was recently alerted that a paper published in the journal in 2015 [1], had been identified in a scoping review listing articles suspected of having breached international ethics guidance by reporting research that used organs or data derived from executed prisoners [2]. The European Society of Organ Transplantation (ESOT) has endorsed the Declaration of Istanbul [3], which unequivocally rejects the use of executed prisoners as organ donors. As the official journal of ESOT, Transplant International is committed to upholding this ethical principle, and its policies state that manuscripts whose data derives from transplants involving organs obtained from executed prisoners are not eligible for publication [4].

The article reports data from emergency liver transplants performed at the First Affiliated Hospital of Sun Yat-Sen University in Guangzhou, People’s Republic of China, between 2006 and 2010. While the use of organs from executed prisoners was permitted under national policy in China at the time of the study, this practice does not comply with international ethical guidelines. The article does not provide information regarding the origin of donors, causes of death, or consent for donation, meaning the donor source cannot be verified. An investigation was conducted in accordance with Transplant International policies. The authors and their institution have remained unresponsive to multiple formal requests to provide donor details and explicit verification that executed prisoners were not utilized. This is a breach of Transplant International guidelines and those of the Committee on Publication Ethics (COPE). Consequently, given the inability to verify the ethical sourcing of the organs, the article has been retracted.

This retraction was approved by the Editor-in-Chief of Transplant International. The authors have not responded to correspondence regarding this retraction.
